# Circular RNAs: New Epigenetic Signatures in Viral Infections

**DOI:** 10.3389/fmicb.2020.01853

**Published:** 2020-07-31

**Authors:** Javid Sadri Nahand, Sogol Jamshidi, Michael R. Hamblin, Maryam Mahjoubin-Tehran, Massoud Vosough, Marzieh Jamali, Alireza Khatami, Mohsen Moghoofei, Hossein Bannazadeh Baghi, Hamed Mirzaei

**Affiliations:** ^1^Department of Virology, Faculty of Medicine, Iran University of Medical Sciences, Tehran, Iran; ^2^Student Research Committee, Iran University of Medical Sciences, Tehran, Iran; ^3^Wellman Center for Photomedicine, Massachusetts General Hospital, Boston, MA, United States; ^4^Department of Dermatology, Harvard Medical School, Boston, MA, United States; ^5^Laser Research Centre, Faculty of Health Science, University of Johannesburg, Doornfontein, South Africa; ^6^Student Research Committee, Mashhad University of Medical Sciences, Mashhad, Iran; ^7^Department of Medical Biotechnology, Faculty of Medicine, Mashhad University of Medical Sciences, Mashhad, Iran; ^8^Department of Regenerative Medicine, Cell Science Research Center, Royan Institute for Stem Cell Biology and Technology, ACECR, Tehran, Iran; ^9^Department of Gynecology and Obstetrics, Mahdieh Hospital, Shahid Beheshti University of Medical Sciences, Tehran, Iran; ^10^Department of Microbiology, Faculty of Medicine, Kermanshah University of Medical Sciences, Kermanshah, Iran; ^11^Infectious and Tropical Diseases Research Center, Tabriz University of Medical Sciences, Tabriz, Iran; ^12^Immunology Research Center, Tabriz University of Medical Sciences, Tabriz, Iran; ^13^Research Center for Biochemistry and Nutrition in Metabolic Diseases, Kashan University of Medical Sciences, Kashan, Iran

**Keywords:** circular RNA, VcircRNA, viral infection, biomarker, back-splicing, epigenetics

## Abstract

Covalent closed circular RNAs (circRNAs) can act as a bridge between non-coding RNAs and coding messenger RNAs. CircRNAs are generated by a back-splicing mechanism during post-transcriptional processing and are abundantly expressed in eukaryotic cells. CircRNAs can act via the modulation of RNA transcription and protein production, and by the sponging of microRNAs (miRNAs). CircRNAs are now thought to be involved in many different biological and pathological processes. Some studies have suggested that the expression of host circRNAs is dysregulated in several types of virus-infected cells, compared to control cells. It is highly likely that viruses can use these molecules for their own purposes. In addition, some viral genes are able to produce viral circRNAs (VcircRNA) by a back-splicing mechanism. However, the viral genes that encode VcircRNAs, and their functions, are poorly studied. In this review, we highlight some new findings about the interaction of host circRNAs and viral infection. Moreover, the potential of VcircRNAs derived from the virus itself, to act as biomarkers and therapeutic targets is summarized.

## Introduction

Single-stranded circular RNAs (circRNAs) belong to the non−coding RNA family. Unlike linear RNAs, they are take the form of a covalently closed continuous loop with neither 5′ capping nor 3′ polyadenylation, and are formed by a back-splicing process (Guo et al., 2014; Holdt et al., 2018). Sanger et al. (1976) originally discovered the presence of circRNAs in a viroid-infected plant using electron microscopy in 1970. Later, the presence of circRNA was detected in the hepatitis D virus (HDV) and in yeast mitochondria (Arnberg et al., 1980). The first study to confirm the presence of circRNAs in human cells by Nigro et al. (1991), reported the detection of circular transcripts derived from the tumor suppressor gene DCC in several human tumor cell lines. Recently, with the advent of new sequencing technologies, such as next-generation sequencing (NGS), a growing number of circRNAs have been reported (Hanan et al., 2017; Wang et al., 2017; Zhang Z.-C. et al., 2018; Zaiou, 2019), and have now become a “hot topic.”

Since some circRNAs have the ability to encode proteins, they have therefore been suggested as a crucial bridge between non-coding RNAs and coding RNAs (Braicu et al., 2019). To date, the function(s) of several circRNAs have been identified, including the sponging of microRNA (miRNA), regulation of RNA transcription and protein production, and the translation of proteins and peptides (Li J. et al., 2015). Considering the multifunctional nature of circRNAs, they may be involved in many biological and pathological processes, which could influence the progression of diseases such as cancer and viral infections. In cancer it has been suggested that circRNAs could affect the malignant phenotype through regulating cancer-related pathways, and could exert either an anti-cancer activity or a pro-cancer activity. Therefore, these molecules could either act as tumor suppressors or alternatively as oncogenes depending on the tumor type and stage (Wang et al., 2017), and could therefore serve as a therapeutic target in the treatment of cancer.

Moreover, the unique covalent closed-loop structure of circRNAs makes them insensitive to the enzyme activity of ribonucleases (Harland and Misher, 1988). The expression levels of circRNAs are generally lower than those of messenger RNAs (mRNAs) (Jeck et al., 2013; Guo et al., 2014; Shang et al., 2019). However, circRNAs mostly act in a tissue and cell type-specific manner, and have been found to be stably expressed in several biological materials including saliva, tissue, blood, and exosomes. Therefore, circRNAs could be potential biomarkers in the diagnosis and prognosis of several different diseases (Zhang Z. et al., 2018; Naeli et al., 2019). Up to now, only relatively few studies have been conducted on the interaction between viruses and host circRNAs; however, it has been reported that the expression patterns of host circRNAs are altered in virus-infected cells and patients compared to the control groups (Cui et al., 2018; Shi et al., 2018; Zheng et al., 2018; Yu T. et al., 2019). Therefore, it has been proposed that viruses are likely to use these molecules for their own progression. It has also been observed that some viral genes are able to produce viral circRNA (VcircRNA) molecules through back-splicing (Toptan et al., 2018; Ungerleider et al., 2019; Zhao et al., 2019b), but the viral genes that can encode circRNAs and their functions are poorly studied. In this review, we will first briefly discuss the biogenesis, and function of circRNAs, and then highlight some new findings concerning the interaction of host circRNAs with viral infections. Lastly, circRNAs derived from the virus genome and their potential as biomarkers or therapeutic targets for viral diseases are summarized.

## The Biogenesis of circRNAs

**FIGURE 1:**
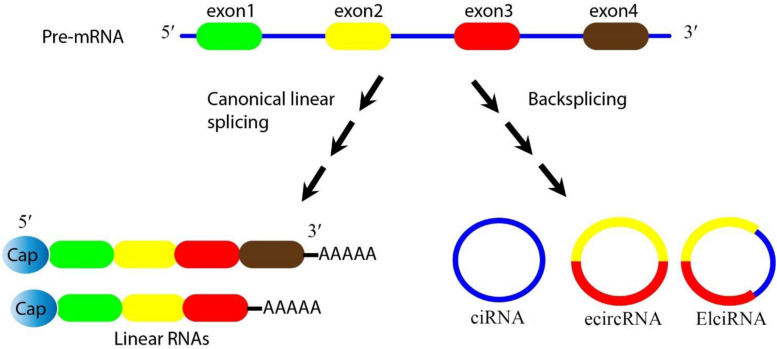
Biogenesis of circRNAs. Pre-mRNA can be processed through either canonical splicing to produce linear RNAs **(left)** or backsplicing to generate circRNAs **(right)**. CircRNAs are mainly divided into three categories based on their components. Exonic circRNAs (ecircRNAs) are exclusively composed of exons and represent the largest group of circRNAs, intronic circRNA (ciRNA) are exclusively composed of introns, and exon-intron circRNA (elciRNA) are exon-intron sequences. In this figure, arrows show the splicing events for generating the products, blue lines show introns, and colored lines (red/yellow) show exons.

In eukaryotic cells, the splicing of precursor mRNA (pre-mRNA) is catalyzed by spliceosomes. The spliceosome is an assembly of small nuclear ribonucleoproteins (snRNPs) and other protein factors that act to join together exons, and remove introns (Valadkhan, 2005). During splicing, if the upstream 5′ splice-site (donor site) is joined to the downstream 3′ splice site (acceptor site) this leads to the production of linear (m)RNAs. However the spliceosome can also generate circRNAs during the processing of pre-mRNA, pre-transfer ribonucleic acid (tRNA), and pre-ribosomal ribonucleic acid (rRNA) molecules (Zhang et al., 2017). These loop structures can originate from exons or from introns in either a single sequence or two different sequences (Xin et al., 2017). CircRNAs are considered to be a highly stable class of long non-coding RNAs, and are believed to be an important bridge between non-coding RNAs and coding RNAs (Braicu et al., 2019). CircRNAs are generally divided into three categories based on their components. Firstly, exonic circRNAs (ecircRNAs) are exclusively composed of exons, and represent the largest group of circRNAs. Secondly, intronic circRNAs (ciRNA) are exclusively composed of introns. Thirdly, exon-intron circRNAs (elciRNA) contain both exon and intron related sequences (Figure 1) (Wang et al., 2019). Intergenic circRNA is another non-exonic circRNA identified by a circRNA identifier (CIRI). This integrated circRNA is formed by two intronic circRNA fragments (ICFs) flanked by GT-AG splicing signals acting as the splice donor (SD) and splice acceptor (SA) sites of the circular junction (Geng et al., 2018). Studies using different techniques have shown that ecircRNAs are localized within the cytoplasm (Jeck and Sharpless, 2014). On the other hand, ciRNA and elciRNA are predominantly localized within the nucleus (Meng et al., 2017). The “direct back-splicing” and “lariat formation” are the two main mechanisms that can lead to the conversion of pre-mRNAs into circRNAs (Qu et al., 2015; Yang et al., 2017; Braicu et al., 2019). In the back splicing process, the downstream 5′ donor site is linked to the upstream 3′ acceptor site resulting in the production of circRNAs (Kristensen et al., 2019; Zhao et al., 2019c). Interestingly, multiple circRNAs can be generated through two different types of back-splicing, including alternative 5′ back-splicing (A5BS), and alternative 3′ back-splicing (A3BS). A5BS occurs when two or more 5′ downstream back-splice sites bind to the same upstream 3′ back-splice site in a reverse orientation. Similarly, A3BS occurs when two or more upstream 3′ back-splice sites bind to the same downstream 5′ back-splice site (Qu et al., 2015; Dong et al., 2018).

The two different types of the lariat formation mechanism, are “exon skipping” and “intron−pairing−driven” circularization (Wang et al., 2015). In intron−pairing−driven circularization, the 5′ end of the pre-mRNA is cleaved by the U1 small nuclear RNA (snRNA), and the 5′- and 2′-bases between the guanidine and adenosine are ligated together. The processed intronic lariat is retained in a circular form within the nucleus (Konarska et al., 1985; Hansen et al., 2013; Braicu et al., 2019). Exon skipping occurs naturally only at a low frequency (Goyenvalle et al., 2004). In exon skipping a “hetero-lariat” is formed that contains both introns and exons (Suzuki et al., 2016; Zhu et al., 2017; Braicu et al., 2019). This process could continue until all the introns have been removed, and only circRNAs containing exonic sequences are formed (Yang et al., 2017).

Some studies have shown that various viruses can encode circRNAs by alternative splicing of the viral mRNAs (Bodescot and Perricaudet, 1986; Purcell and Martin, 1993). Thus, viruses can produce VcircRNAs (Zhao et al., 2019b) using a back-splicing mechanism (Toptan et al., 2018). However, only few studies have been performed on this topic and more experimental studies are needed.

## The Functions of circRNAs

Considering the great diversity of circRNAs and their many individual targets, it is to be expected that circRNAs will play several key roles within the cell, but not all of them have yet been well characterized. However, some cellular and molecular roles of circRNAs have been identified (Ruskin and Green, 1990; Vicens and Westhof, 2014). As mentioned above, circRNAs can be derived from introns (intronic circRNA or ciRNA), exons (exonic circRNA or ecircRNA) and exon-intron sequences (exon-intron circRNA or elciRNA) (Zhang et al., 2013; Zhang et al., 2014; Chen et al., 2015; Li Z. et al., 2015). Some elciRNAs and ciRNAs are found in the nucleus and are likely to play a regulatory function in gene transcription, whereas ecircRNAs are predominantly found in the cytoplasm, and are involved in post-transcriptional regulation (Haddad and Lorenzen, 2019). The structural examination of some circRNAs has shown that they contain N6-methyladenosine modifications or an internal ribosome entry site (IRES), and have the potential to be efficiently translated into proteins (Yu and Kuo, 2019). Depending on the type of protein produced, they could play important roles within the cell. For example, Yang et al. (2019) found that circ-FBXW7 was abundantly expressed in the normal human brain tissue, and reported that circ-FBXW7 encodes the FBXW7-185aa protein. The FBXW7-185aa protein inhibits cell proliferation and tumorigenesis, while its down-regulation is associated with the induction of a malignant phenotype in cancer cells (Yang et al., 2019). Additionally, circRNAs may also affect the activity of proteins, and are probably involved in the modulation of protein–protein interactions, protein binding, and protein sorting (Du et al., 2017). For example, circRNA CDR1 interacts strongly with argonaute (AGO) proteins, which are essential components of the RISC complex (Memczak et al., 2013).

CircRNAs could also regulate the activity of miRNAs via a novel mechanism (Kulcheski et al., 2016). Endogenous circRNAs can function as miRNA sponges, to negatively regulate the activity of miRNAs by removing them from their site of action (Hansen et al., 2013; Memczak et al., 2013). Since miRNAs control many biological events, circRNAs could influence these processes through a miRNA sponging effect (Kulcheski et al., 2016). It has been shown that several miRNA sponges may be associated with human disease, such as Alzheimer’s, Hirschsprung’s, diabetes, osteoarthritis, and several types of cancer (Lukiw, 2013; Wang et al., 2016; Zhao et al., 2016; Han et al., 2017; Peng et al., 2017; Zhao et al., 2017; Zhou and Yu, 2017; Zhou Z.-B. et al., 2018; Kristensen et al., 2018).

For example, it has been shown that some circRNAs can contribute to cancer progression by sponging tumor-suppressive miRNAs, or alternatively suppress the cancer phenotype via sponging of oncogenic miRNAs. It has generally been observed that tumor suppressor circRNAs are down-regulated in cancers, while oncogenic circRNAs are up-regulated. For instance, Hao et al. (2019) reported that circ_0007534 was over-expressed in patients with pancreatic ductal adenocarcinoma. They also found that ectopic expression of circ_0007534 caused increased proliferation, invasion, and migration in the PDAC cell line. This oncogenic effect of circ_0007534 was mediated by sponging of miR-892b and miR-625 (Hao et al., 2019). Circ_0026344 was found to be down-regulated in colorectal cancer, while the expression level of miR-31 and miR-21 was increased in colorectal cancer tissue. Further analysis demonstrated that over-expression of circ_0026344 decreased tumor growth and increased apoptosis in cell lines, through sponging of miR-21 and miR-31 (Yuan et al., 2018).

**FIGURE 2:**
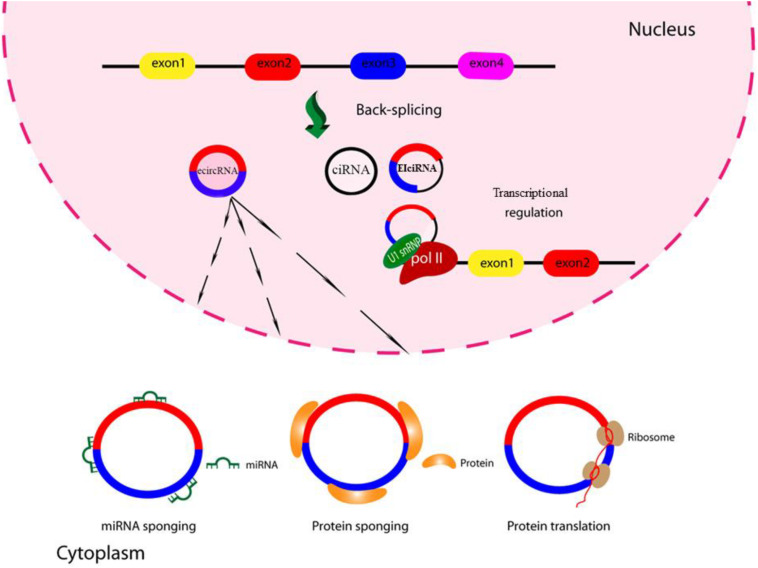
Schematic of circRNA functions. CircRNAs might function as miRNA sponges by competing for the binding of miRNA sequences, lessening the impact of miRNA-mediated regulation of gene expression. CircRNAs might function as protein sponges. Some circRNAs might control the expression of proteins by sequestering mRNA translation start sites. CircRNAs might be translated to create functional proteins.

However, up to now only a few studies have been performed on the function of circRNAs in viral infections, and the role they may play in inhibiting or enhancing virus replication is not well understood. A summary of the circRNA functions is shown in Figure 2.

## Host circRNAs and Viral Infections

As discussed above, it has been shown that the deregulation of circRNA expression in cancer cells, i.e., down-regulation or up-regulation, can contribute to the malignancy of cancer cells (Wu et al., 2019). However, it is not yet clear whether the aberrant expression of circRNAs in virus-infected cells, ultimately contributes to viral replication or not. It has been demonstrated that viral infections can remodel the entire transcriptome landscape of host cells (Park et al., 2015; Hu et al., 2016). One important effect is to improve the viral replication cycle through modifying transcriptomic responses related to antiviral immunity and cellular apoptosis. This common strategy has been observed in many different viral infections (Gao et al., 2017a; Martin et al., 2017; Wang et al., 2017). HSV-1 infection, in addition to altering the expression of cellular genes, can affect RNA processing in the host cells leading to changes in alternative polyadenylation and splicing in the host transcriptome (Zheng et al., 2017; Shi et al., 2018). An interaction between viruses and circRNAs was observed in a study by Li et al. (2017). They reported that the transcription factor NF90/NF110 (derived from interleukin enhancer binding factor, ILF3) could regulate the biogenesis and function of circRNAs. Upon viral infection, these factors led to the down-regulation of circRNA levels (Li et al., 2017).

Recently, Shi et al. (2018) examined the profile of the circRNA transcriptome in HSV-infected cells. In this study, the aberrant circRNA expression profile in the HSV-1-infected KMB17 cell line was investigated using deep RNA sequencing. The results showed that the expression levels of 536 separate circRNAs were significantly dysregulated after HSV-1 infection, and of these, 348 circRNAs were down-regulated and 188 were up-regulated. The expression levels of five circRNAs (circRNA7231, circRNA3683, circRNA3046, circRNA6783, and circRNA7752) were measured by real time quantitative reverse transcription (RT-qPCR), and it was found that their expression was significantly increased after infection with HSV-1 (Shi et al., 2018). However, the function and interaction of these circRNAs with the HSV-1 virus, and whether they could be used as diagnostic biomarkers or therapeutic targets is still unclear.

Cervical cancer is the fourth most common cancer among women throughout the world (Bhadelia, 2019). Persistent infection with the high risk type of human papillomaviruses (HPV-16 and HPV-18) is the main cause of the progression of papilloma lesions to cervical cancer, and is found in more than 70% of cases (Ghittoni et al., 2010). HPV E6 and E7 oncoproteins play a critical role in the transformation of cervical cells by interfering with the p53 and pRb pathways, respectively (Burroni et al., 2013). In addition, HPV E6 and E7 oncoproteins can modulate the expression of cellular miRNAs, which may contribute to the tumorigenesis of cervical cancer cells (Nahand et al., 2019). Since circRNAs act as sponges for miRNAs, the virus is likely to indirectly alter the expression level of miRNAs by affecting circRNAs. Current studies suggest that circRNAs have the potential to be used as diagnostic biomarkers in cervical cancer (Chaichian et al., 2019). Gao et al. (2017b) demonstrated that hsa_circ_0018289 was over-expressed in cervical cancer cells, and knockdown of this circRNA could suppress the migration, proliferation, and invasion of cervical cancer cells. They suggested hsa_circ_0018289 may play an oncogenic role in the tumorigenesis of cervical cancer (Gao et al., 2017b).

One study evaluated the circRNA expression profile in cervical cancer cells infected with HPV16 E7. Using a microarray approach, they found that 526 separate circRNAs had significantly altered expression levels (174 down-regulated 352 up-regulated) (Zheng et al., 2018). In order to confirm these results, eight circRNAs with different expression levels were measured using the qRT-PCR method, and it was found that the results of both methods were in agreement. The finding that the expression pattern of circRNAs in HPV-16 E7 infected cells was altered (Zheng et al., 2018), provided new insights into potential therapeutic targets and candidate biomarkers in cervical cancer associated with HPV.

MicroRNAs are involved in many cellular biological processes, including inflammation, cellular differentiation, apoptosis, and also in viral infection (Hagen and Lai, 2008; Hulsmans and Holvoet, 2013; Sadri et al., 2019). Transmissible gastroenteritis virus (TGEV) is a member of the Coronavirus family with a positive-sense single-stranded RNA genome, which emerged as a cause of severe gastroenteritis in pigs (Laude et al., 1990). TGEV can activate the NF-κB pathway resulting in severe inflammation in the epithelial cells of porcine intestines (Ma et al., 2014). Ma et al. (2018) analyzed the expression profile of circRNAs, miRNAs, and mRNAs in TGEV-infected IPEC-J2 (intestinal porcine epithelial cell-jejunum 2) cell line using NGS technology. They observed that miR-22 was over-expressed in TGEV infected cells, and suggested that ssc_circ_009380 by sponging of miR-22 was able to trigger NF-κB pathway activity. It is known that miR-22 exerts its anti-inflammatory effects *via* targeting IL-6, CCL5 and DDX58 (Ma et al., 2018). Moreover, it was demonstrated that miR-22 could attenuate myocardial ischemia-reperfusion injury in rats via an anti-inflammatory mechanism (Yang et al., 2016).

The hepatitis B virus (HBV) belongs to the Hepadnaviridae family, and between 5 and 10% of HBV-infected patients develop a chronic liver infection about 6 months after the acute infection (Mayerat et al., 1999). Chronic HBV infection can cause serious liver diseases, such as cirrhosis and hepatocellular carcinoma (HCC) (Sinn et al., 2015). The progression of HBV infection in many individuals is limited by the host immune response. However, in others the infections becomes chronic due to comparatively weak T-cell responses against HBV, suppression of NK cell function by several immunomodulatory cytokines, and ineffective adaptive responses against HBV viral infected cells (Mayerat et al., 1999; Tan et al., 2015). Other factors, such as alcohol, aflatoxin, gut microbiota, mannose-binding lectin (MBL) genotype, and also miRNAs, can affect the progression of chronic hepatitis B (Ohnishi et al., 1982; Handin et al., 2003; Chong et al., 2005; Sagnelli et al., 2018; Sadri Nahand et al., 2019). Evidence suggests that some circRNAs are associated with the carcinogenesis process of HCC (Fu et al., 2018). Zhu et al. (2018) showed that hsa_circ_0067934 by sponging of miR−1324, could alter the FZD5/Wnt/β−catenin signaling pathway, which was involved in migration, proliferation, and invasion of HCC cells. However, the role of HBV infection in altering the expression of circRNAs, which may then contribute to liver disease, is poorly characterized. Yang et al. (2016, 2017, 2019) investigated the deregulation of circRNAs in hepatitis B-related HCC, and found that circRNA_100338 was significantly over-expressed in HCC tissue. They also found that circRNA_100338 could act as a miR-141-3p sponge in HCC tissue, and suggested that circRNA_100338 could be a new biomarker for the diagnosis of HBV-associated HCC (Huang et al., 2017). Yu J. et al. (2019) examined the expression of circRNAs in plasma and HCC tissues from infected patients by microarray and qRT-PCR techniques. They reported that there was a positive correlation between the expression levels of hsa_circ_0139897, hsa_circ_0000976, and hsa_circ_0007750 in the patient samples, and they were significantly increased after hepatectomy (Yu J. et al., 2019). Data concerning the role of circRNAs as diagnostic or therapeutic biomarkers in HBV-HCC is limited and needs further investigation.

Autophagy is a form of programmed cell death, and is a highly conserved cellular process designed to dispose of damaged cellular organelles or protein aggregates (Pourhanifeh et al., 2020). Autophagy is activated in many viral infections such as HSV-1, HBV, and influenza A virus (IAV). Autophagy has been reported to either inhibit or stimulate the replication of some viruses, and plays a critical role in modulating cell survival (Ahmad et al., 2018). Additionally, it has been observed that viral proteins and noncoding RNAs produced during viral infections, can regulate autophagy in the host cells, which may contribute to escape from the immune system, release of viruses from cells, and viral replication (Kudchodkar and Levine, 2009; Fu et al., 2015; Wu et al., 2016). circRNAs have the ability to either attenuate or activate autophagy depending on the conditions (Du et al., 2018; Zhou L.-Y. et al., 2018), and therefore viruses may also affect autophagy by altering the expression of circRNA. Recently, it was reported that the expression level of circ-GATAD2A was up-regulated during infection of A549 cells with IAV. Furthermore, after the knockdown of circ-GATAD2A within the cells, H1N1 replication was suppressed and autophagy was promoted. In agreement, the up-regulation of circ-GATAD2A in A549 cells-infected with H1N1 promoted virus replication and also inhibited autophagy. Overall, the results of this study suggested that this circRNA could promote H1N1 replication by suppressing autophagy (Yu T. et al., 2019), and this finding might be useful for therapeutic purposes.

The role of circRNAs in viral replication has only so far been studied to a limited extent, however, given the significant changes observed in the circRNA expression profile during viral infections, we believe that viruses are likely to use this effect to their advantage. Reports of the deregulation of circRNAs during viral infections are shown in Table 1.

**TABLE 1:** Cellular circRNAs and viral infections.

**circRNA**	**Virus**	**Dysregulation**	**Model**	**Type of cell line**	**References**
hsa_circ_0003046 (circRNA3046)	HSV-1	Up	*In vitro*	KMB17	Shi et al. (2018)
hsa_circ_0003683 (circRNA3683)	HSV-1	Up	*In vitro*	KMB17	Shi et al. (2018)
hsa_circ_0007752 (circRNA7752)	HSV-1	Up	*In vitro*	KMB17	Shi et al. (2018)
hsa_circ_0007231 (circRNA7231)	HSV-1	Up	*In vitro*	KMB17	Shi et al. (2018)
hsa_circ_0006783 (circRNA6783)	HSV-1	Up	*In vitro*	KMB17	Shi et al. (2018)
hsa_circ_0051620	HPV-16 (E7)	Up	*In vitro*	C33A, CaSKi cells	Zheng et al. (2018)
hsa_circ_0052602	HPV-16 (E7)	Up	*In vitro*	C33A, CaSKi cells	Zheng et al. (2018)
hsa_circ_0005389	Chronic hepatitis B	Up	Human	-	Zhou T. C. et al. (2018)
hsa_circ_0000038	Chronic hepatitis B	Up	Human	-	Zhou T. C. et al. (2018)
hsa_circ_0100381	HBV-related HCC	Up	Human	-	Wang et al. (2018)
hsa_circ_0103489	HBV-related HCC	Up	Human	-	Wang et al. (2018)
hsa_circ_0104351	HBV-related HCC	Up	Human	-	Cui et al. (2018)
hsa_circ_0102814	HBV-related HCC	Up	Human	-	Cui et al. (2018)
hsa_circ_0103489	HBV-related HCC	Up	Human	-	Cui et al. (2018)
hsa_circ_0102109	HBV-related HCC	Up	Human	-	Cui et al. (2018)
hsa_circ_0100381	HBV-related HCC	Up	Human	-	Cui et al. (2018)
hsa_circ_0027089	HBV-related HCC	Up	Human (plasma)	-	Zhu et al. (2019)
hsa_circ_0000976	HBV-related HCC	Up	Human (tissue and plasma)	Huh-7, Hep-G2	Yu J. et al. (2019)
hsa_circ_0007750	HBV-related HCC	Up	Human (tissue and plasma)	Huh-7, Hep-G2	Yu J. et al. (2019)
hsa_circ_0139897	HBV-related HCC	Up	Human (tissue and plasma)	Huh-7, Hep-G2	Yu J. et al. (2019)
hsa_circ_0030753 (circ-GATAD2A)	Influenza (H1N1)	Up	*In vitro*	A549 cells	Yu T. et al. (2019)
hsa_circ_0001400	KSHV	Up	*In vitro*	HUVEC, 293T cells	Tagawa et al. (2018)
hsa_circ_0001741	KSHV	Up	*In vitro*	HUVEC, 293T cells	Tagawa et al. (2018)
chi_circ_7880 (circRNA7880)	ORFV	Up	*In vitro*	GSF cells	Pang et al. (2019)
hsa_circ_0048867	HPV-16 (E7)	Down	*In vitro*	C33A, CaSKi cells	Zheng et al. (2018)
hsa_circ_0038475	HPV-16 (E7)	Down	*In vitro*	C33A, CaSKi cells	Zheng et al. (2018)
hsa_circ_0035918	HPV-16 (E7)	Down	*In vitro*	C33A, CaSKi cells	Zheng et al. (2018)
hsa_circ_0056353	HPV-16 (E7)	Down	*In vitro*	C33A, CaSKi cells	Zheng et al. (2018)
hsa_circ_0026527	HPV-16 (E7)	Down	*In vitro*	C33A, CaSKi cells	Zheng et al. (2018)
hsa_circ_0037213	HPV-16 (E7)	Down	*In vitro*	C33A, CaSKi cells	Zheng et al. (2018)
hsa_circ_0102904	HBV-related HCC	Down	Human	-	Cui et al. (2018)
hsa_circ_0001225	HBV-related HCC	Down	Human	-	Cui et al. (2018)
hsa_circ_0101092	HBV-related HCC	Down	Human	-	Cui et al. (2018)
hsa_circ_0101764	HBV-related HCC	Down	Human	-	Cui et al. (2018)
hsa_circ_0100327	HBV-related HCC	Down	Human	-	Cui et al. (2018)
hsa_circ_0000650	Chronic hepatitis B	Down	Human	-	Zhou T. C. et al. (2018)
hsa_circ_0101764	HBV-related HCC	Down	Human	-	Wang et al. (2018)
ssc_circ_0009380 (circEZH2)	TGEV	Down	*In vitro*	IPEC-J2	Ma et al. (2018), Zhao et al. (2019d)
chi_circ_1001 (circRNA1001)	ORFV	Down	*In vitro*	GSF cells	Pang et al. (2019)
chi_circ_1684 (circRNA1684)	ORFV	Down	*In vitro*	GSF cells	Pang et al. (2019)
chi_circ_3127 (circRN3127)	ORFV	Down	*In vitro*	GSF cells	Pang et al. (2019)
mmu_circ_001273 (circRNA1273)	SV40	-	*In vitro*	AGMK-derived Vero cells	Shi et al. (2017)
mmu_circ_001040 (circRNA1040)	SV40	-	*In vitro*	AGMK-derived Vero cells	Shi et al. (2017)
mmu_circ_001005 (circRNA1005)	SV40	-	*In vitro*	AGMK-derived Vero cells	Shi et al. (2017)
mmu_circ_001013 (circRNA1013)	SV40	-	*In vitro*	AGMK-derived Vero cells	Shi et al. (2017)
mmu_circ_001220 (circRNA1220)	SV40	-	*In vitro*	AGMK-derived Vero cells	Shi et al. (2017)
mmu_circ_001088 (circRNA1088)	SV40	-	*In vitro*	AGMK-derived Vero cells	Shi et al. (2017)
mmu_circ_001195 (circRNA1195)	SV40	-	*In vitro*	AGMK-derived Vero cells	Shi et al. (2017)

## Virus-Encoded circRNAs

Viral infections in both humans and animals have been reported to occur with increased frequency in recent years. There are several types of viral disease, according to the underlying virus. HDV was the first human viral pathogen to be found to possess a circRNA within the genome (Kos et al., 1986). This circRNA can encode only one protein, hepatitis delta antigen (HDAg) (Farci, 2003). The *Herpesviridae* is a large family of DNA viruses, which can be categorized into three subfamilies: Alphaherpesvirinae, e.g., herpes simplex virus (HSV)-1, 2, varicella-zoster virus (VZV), Betaherpesvirinae, e.g., cytomegalovirus (CMV), human herpes virus (HHV)-6, 7, and Gammaherpesvirinae, e.g., Epstein Barr virus (EBV) and Kaposi’s sarcoma-associated herpes virus (KSHV). Almost all herpes viruses remain latent after the primary infection has subsided and are later reactivated under certain conditions (Pellett and Roizman, 2013). The viral replication and the expression of viral genes in the latent phase are limited, and this accounts for the ability of the virus to escape from the host immune response (Grinde, 2013). HPV is another viral infection that is passed between people through skin-to-skin contact. As discussed earlier, persistent infection with HPV-16 and HPV-18 is the main cause of the papilloma lesions that are precursors to cervical cancer, and is found in more than 70% of cases (Ghittoni et al., 2010). Anal squamous cell carcinoma (ASCC) is a rare malignancy, but high-risk strains of HPV have been implicated in 70–90% of ASCC cases (Martin et al., 2018).

Hepatitis D virus produces its circRNA molecules by using the cellular machinery and the rolling-circle mechanism. The connection of both ends of the RNA molecules by formation of 3′–5′ or 2′–5′ phosphodiester bonds leads to the formation of circRNA molecules (Reid and Lazinski, 2000; Eger et al., 2018). Two advantages of the circularization process of the HDV genome or viral encoded circRNAs could be suggested: (a) escape from recognition via innate immune pattern recognition receptors such as RIG-I and MDA-5; and (b) protection against degradation by intracellular exonucleases (Eger et al., 2018).

The innate immune system, the first line of host defense, is very important against pathogens. It contains pattern recognition receptors which respond to some specific structures that are typical of pathogens (Mogensen, 2009). Protein kinase R (PKR) is one of the receptors that recognize long dsRNAs (>33 bp) in the cytoplasm, and inhibits synthesis of protein. Although PKR should be readily activatable if needed, it should remain in an inactive state to prevent autoimmunity and inappropriate reactions. Studies have shown that activation of PKR can be inhibited by binding to the adenovirus small-noncoding VAI RNA or short (16–33 bp) dsRNAs. Liu C. X. et al. (2019) found that endogenous circRNAs could bind to PKR (Kitajewski et al., 1986; Zheng and Bevilacqua, 2004). Importantly, by comparing the binding profiles of circular and linear RNAs with the same base sequence, they found that circRNAs could bind more strongly to PKR than linear RNAs. This result suggested that the secondary structures of circRNAs and linear RNAs are different. In fact, structural mapping showed that circRNAs inside cells could form stable secondary structures which contained short (16–26 bp) imperfect duplexes, whereas the linear RNAs were folded into unstable and more dynamic structures (Liu C. X. et al., 2019).

As mentioned above, alternative splicing is a key post-transcriptional mechanism involved in the production of circRNA molecules from pre-mRNAs (Eger et al., 2018). One way that the virus could produce multiple products from a single gene, is alternative splicing. Since this mechanism occurs in the nucleus, DNA viruses (e.g., *Adenoviridae*, *Herpesviridae*, *Papillomaviridae*, *Polyomaviridae*, and *Hepadnaviridae*) (Ge and Manley, 1990; Tormanen et al., 2006; Verma and Swaminathan, 2008; Mole et al., 2009; Li et al., 2010; Guan et al., 2011) and some RNA viruses (e.g., *Retroviridae*, *Bornaviridae*, and *Orthomyxoviridae*) (Hope, 1999; Tomonaga et al., 2000; Robb and Fodor, 2012) that all replicate within the nucleus, may be capable of producing circRNAs. Recently, the RNA-sequencing technique (RNA-seq) has been used to identify the expression of circRNAs, and many results have been confirmed by RT-qPCR (Li and Han, 2019). RNA-seq was applied to evaluate the expression level of RNAs, and to survey the overall RNA population, including long noncoding RNA (lncRNA), small RNA, rRNA, and circRNA (Wang et al., 2009; Ingolia et al., 2012). The following methods have employed for different purposes in the RNA-seq experiments: (a) RiboMinus treatment was used for the accurate comparison between circRNA and mRNA expression levels; (b) RNase R and RiboMinus treatment was used for the comprehensive profiling of circRNAs; and (c) poly(A) enrichment was used to increase the concentrations of lncRNA and mRNA (Ji et al., 2019).

According to the different expression patterns of EBV genes, at least three distinct latent phases (I, II, and III) have been identified (Münz, 2015). In addition to its coding RNAs, the EBV virus can also express non-coding RNAs. It has been shown that the intronic regions of the Bam HI-A region rightward transcript (BART) gene of EBV is capable of expressing many non-coding RNAs (ncRNA), including the small non-coding EBV-encoded RNA 1 (EBER1) and EBER2 (Fok et al., 2006; Verhoeven et al., 2019). Unlike EBV, differential gene expression in various types of Karposi’s sarcoma tumor has been described (Dittmer and Damania, 2013). During latent infection with KSHV a number of non-coding RNAs have been detected, including miRNAs and the polyadenylated nuclear RNA (PAN) (Samols et al., 2007; Conrad, 2016; Toptan et al., 2018). PAN transcripts are important for KSHV replication and are abundantly expressed during lytic replication, but are found only in low levels during the latent phase (Conrad, 2016). The viral genes encoding circRNAs in many viruses have not yet been fully investigated.

Toptan et al. (2018), for the first time investigated the VcircRNAs encoded by EBV and KSHV in tumor samples and cell lines. In this study RNA-seq was used to identify VcircRNAs after treatment with RNase R. RNase R-resistant RNA sequencing showed that both EBV and KSHV could encode several circRNAs. They observed that in EBV-positive PTLD samples, there were several EBV-derived circRNAs (circBARTs). These were both exon-intron and exon-only circRNAs, formed by back-splicing of BART transcripts. Similarly, circBARTs derived from BART are expressed in all types of latent EBV infection. The authors found that circBARTs were found in all tumors associated with EBV, and suggested that circBARTs could play a role in the reproductive ability of EBV-positive tumor cells. Furthermore, the sequencing of circRNAs within KSHV-infected primary effusion lymphoma (PEL) cells showed that KSHV circRNAs (including circvIRF4 and circPAN/K7.3) were expressed in these cells, and were found to originate from the vIRF4 locus and the PAN region, respectively (Toptan et al., 2018). These VcircRNAs may provide a novel approach for diagnostic biomarkers and therapy for KSHV and EBV associated malignancies.

**FIGURE 3:**
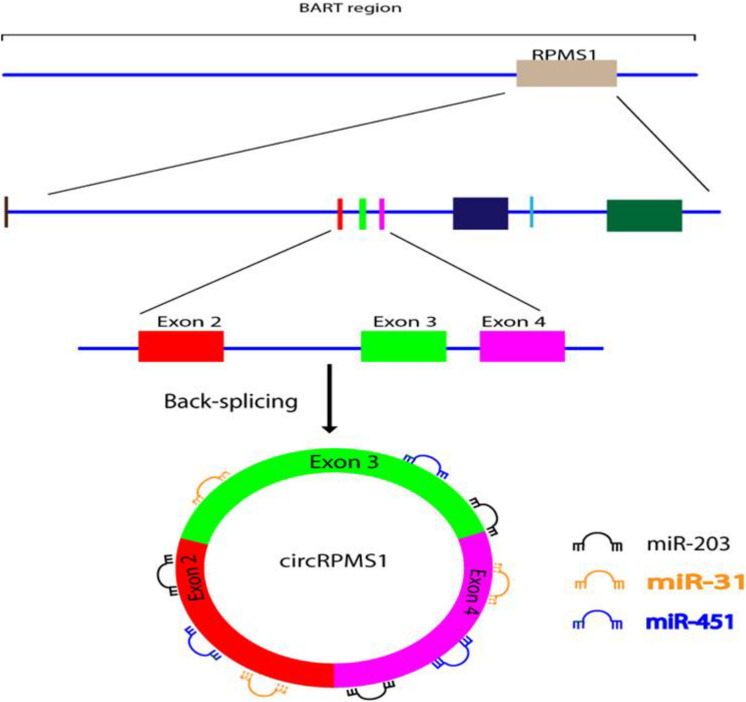
Formation and function of ebv-circRPMS1in EBV-infected NPC cells. Genomic location of the RPMS1 gene in EBV-BART region and ebv-circRPMS1 that originated from exon 2–4 of the RPMS1 gene by back-splicing. CircRPMS1 may be involved in the oncogenesis of NPC via sponging of miR-31, miR-451, and miR-203.

Huang et al. (2019) used RNA-seq to investigate whether EBV is able to encode circRNAs in EBV-infected cell lines, including Akata (latency I), AGS-EBV (latency I), SNU-719 (latency I), C666-1 (latency II), and EBV-positive nasopharyngeal carcinoma (NPC) tissue samples. They found that ebv-circRPMS1 originated from exons 2–4 of the RPMS1 gene by back-splicing (Figure 3) (Huang et al., 2019). In another study, the role of EBV-encoded circRPMS1 in NPC tumorigenesis was examined (Liu Q. et al., 2019), and it was found that circRPMS1 expression was correlated with a shorter survival time, and was further up-regulated in metastatic NPC samples. Moreover, knockdown of circRPMS1 led to inhibition of proliferation and invasion of NPC cells infected with EBV, and induced apoptosis in these cells. Further investigation suggested that circRPMS1 was most probably involved in inducing the epithelial-mesenchymal transition (EMT) in NPC cells and encouraged oncogenesis via sponging of miR-31, miR-451, and miR-203. Down-regulation of this circRNA inhibited the aggressiveness and slowed down the EMT of NPC cells (Liu Q. et al., 2019). These studies suggest that circRPMS1 may be a potential therapeutic target for EBV-associated NPC.

Alternative splicing and polyadenylation regulate HPV-RNA processing during the primary and later-stages of HPV infection. The polycistronic HPV mRNA transcripts undergo alternative splicing using various SD and acceptor sites (Johansson and Schwartz, 2013). In the HPV-16 genome, splicing between SD site 226 (SD226) and splice acceptor site 409 (SA409) or SA526 leads to the production of E7 mRNAs, and also several truncated E6 transcripts (e.g., E6^∗^I). On the other hand, preservation of the intron between SD226 and SA409 generates mRNAs that express E6 (Sedman et al., 1991; Tang et al., 2006; Johansson and Schwartz, 2013). Recently, circRNAs derived from oncogenic HPV subtypes (HPV-16 and 18) were analyzed using the cancer genome atlas (TCGA) RNA-Seq data. The presence of back-splicing in the HPV-16 integrated cell lines (SiHa, CaSki, and UPCI:SCC154 cells) was investigated by inverse PCR. After treatment with RNase R, HPV-E7 derived circRNA (circE7) was detected in all these cell lines. Moreover, they detected circE7 in cells that had been transformed with HPV-16 using both Northern blotting and inverse RT-PCR, but the analogous HPV-18 circE7 could not be robustly detected using these techniques. Several lines of evidence have suggested that circE7 could be translated into the E7 oncoprotein. The knockdown of circE7 led to a decreased level of E7 oncoprotein in CaSki cells, and limited the growth of cancer cells *in vitro* and tumor xenografts *in vivo* (Zhao et al., 2019b).

Chamseddin et al. (2019) compared the role of circE7 as a diagnostic biomarker in ASCC with already established biomarkers (PD-L1, HPV-ISH, and GLUT1), and the relationship between these biomarkers and the clinical outcome in ASCC patients. The circE7 levels were evaluated by RT-qPCR showing that the high circE7 group (top 50% of patients) had the best overall survival rate, and this was also correlated with low PD-L1 expression, positive HPV-ISH, and a better tumor stage (Chamseddin et al., 2019). Overall, studies have shown that patients with HPV-16 positive tumors with a high level of circE7 show improved survival compared to HPV-16 positive tumors with a low level of circE7 (Chamseddin et al., 2019; Zhao et al., 2019b). Due to its importance, more attention should be given to role of circE7 as a biomarker in cervical cancer. A number of other studies are summarized in Table 2.

**TABLE 2:** Viral circular RNAs (VcircRNAs) in viral infections.

**VcircRNAs**	**Virus**	**Derived from**	**Model**	**Cell line**	**localized in cytoplasm/nucleus**	**Note**	**References**
circvIRF4	KSHV	vIRF4	Human (KSHV-positive PELs) *In vitro*	BCBL1, BC1	*Cytoplasm* and nucleus	-	Toptan et al. (2018)
circPAN/K7.3	KSHV	PAN	Human (KSHV-positive PELs) *In vitro*	BCBL1, BC1	*Cytoplasm* and nucleus	-	Toptan et al. (2018)
circvIRF4	KSHV	vIRF4	*In vitro*	BCBL-1, TIVE, iSLK	-	During latency two isoforms of circvIRF4 were generated in each of these cell line models	Ungerleider et al. (2019)
circBART_1.1	EBV	BART	Human (EBV-positive PTLD) *In vitro*	BC-1	Nucleus	CircBARTs were expressed in all EBV tumor latency stages.	Toptan et al. (2018)
circBART_2.1	EBV	BART	Human (EBV-positive PTLD) *In vitro*	BC-1	Nucleus	CircBARTs were expressed in all EBV tumor latency stages.	Toptan et al. (2018)
circBART_1.2	EBV	BART	Human (EBV-positive PTLD) *In vitro*	BC-1	*Cytoplasm* and nucleus	CircBARTs were expressed in all EBV tumor latency stages.	Toptan et al. (2018)
circBART_2.2	EBV	BART	Human (EBV-positive PTLD) *In vitro*	BC-1	*Cytoplasm* and nucleus	CircBARTs were expressed in all EBV tumor latency stages.	Toptan et al. (2018)
ebv_circ_RPMS1	EBV	RPMS1	EBV-infected cell line	AGS-EBV, SNU-719, Akata, C666-1	*Cytoplasm* and nucleus	EBV_circ_RPMS1 was localized in both cytoplasm and nucleus and may act as a novel viral regulator of host and/or viral gene expression	Huang et al. (2019)
circRPMS1	EBV	RPMS1	Human (NPC tissues) *In vitro*	NP69, C666-1, HNE2	*Cytoplasm* and nucleus	CircRPMS1 was increased in metastatic nasopharyngeal carcinoma (NPC). CircRPMS1 targeted to miR-203, miR-31, and miR451, suggesting that circRPMS1 may function as a sponge to these three miRNAs.	Liu Q. et al. (2019)
circRPMS1_E4_E3a	EBV	RPMS1	Human (EBV positive stomach cancer)	-	Nucleus	-	Ungerleider et al. (2018)
circRPMS1_E4_E2	EBV	RPMS1	Human (EBV positive stomach cancer)	-	Nucleus	-	Ungerleider et al. (2018)
circEBNA_U	EBV, rLCV	EBNA	Rhesus SIV/LCV lymphoma model		-	The expression of ebv circEBNA_U in the type III and I latency B-cell models. In all three lymphoma samples rLCV-encoded circEBNA_U	Ungerleider et al. (2019)
circRPMS1_E4_E3a	EBV	RPMS1	Rhesus SIV/LCV lymphoma model	-	-	rLCV circRPMS1_E5_E3a and EBV circRPMS1_E4_E3a and, have almost 88 and 92% homology	Ungerleider et al. (2019)
circRPMS1_E5_E3a	rLCV	RPMS1	Rhesus SIV/LCV lymphoma model	-	-	rLCV circRPMS1_E5_E3a and EBV circRPMS1_E4_E3a and, have almost 88% and 92% homology	Ungerleider et al. (2019)
circM11_ORF69	MHV68	ORF69 M11	*In vitro*	NIH 3T12	-	-	Ungerleider et al. (2019)
circE7	HPV-16	E7	Human (HPV-positive ASCC)	-	-	The up-regulation of circE7 RNA was significantly associated with enhanced survival in ASCC. CircE7 can be detected in formalin-fixed paraffin-embedded cancer samples.	Chamseddin et al. (2019)
circE7	HPV-16	E7	*In vitro In vivo*	CaSki	Cytoplasm	The translation of HPV16 circE7 can produce E7 oncoprotein that is crucial for the transformed growth of CaSki cervical cancer cells	Zhao et al. (2019a, b)

## Conclusion

The discovery of non-coding RNAs (such as lncRNA, miRNA, and circRNA) has provided better understanding of the mechanisms involved in many physiological and pathological processes, and has made them the focal point of studies concerned with their role in cancer, infectious disease (e.g., viral infections), and autoimmune diseases. It was previously thought that circRNAs were only random errors that occurred during transcription, but today it is known that they are fundamentally involved in the regulation of gene expression, and can affect many biological and pathological processes. However, the entire scope of their functions is still unclear. In addition, it has been observed that the expression of circRNAs is altered under different physiological and pathological conditions, and major changes are significantly associated with the progression of several diseases, and may serve as a promising diagnostic biomarker for diseases including viral infections. Recent studies have confirmed that some host circRNAs are deregulated in viral infections, and suggest that the virus uses this cellular mechanism to its advantage. The fact that members of different viral families are capable of encoding circRNAs, promises new advances in the scientific understanding of the diagnosis of viral diseases. Unfortunately, knowledge about the function, mechanism of formation, and transportation of VcircRNAs, as well as identification of the viral genes that encode circRNAs, is still very limited. However, more research into the function of VcircRNAs is crucial, since distinguishing between the roles of host circRNAs and VcircRNAs in viral infection might provide unique insight in the development of novel therapeutic strategies. In the future, the potential application of VcircRNAs can be probably divided into two categories: one being their use as novel biomarkers for prognosis and diagnosis of viral diseases. The other is the development of VcircRNA-based therapeutic approaches which could be useful for deadly viruses, e.g., Ebola virus and human immunodeficiency virus (HIV), or for viruses which are highly contagious and spread rapidly, e.g., novel coronavirus SARS-CoV2. Furthermore, VcircRNA-based vaccines might represent a highly versatile platform for development of vaccines against other viruses. Therefore, further research on circRNAs (especially VcircRNA) could open a new window of opportunity for the treatment of deadly diseases, preventing thousands of deaths and saving a lot of money.

## Author Contributions

HM and HB contributed in conception, design, statistical analysis, and drafting of the manuscript. JN, SJ, MJ, MM-T, MV, AK, and MM contributed in data collection and manuscript drafting. MH critically revised the manuscript. All authors approved the final version for submission.

## Conflict of Interest

MH declares the following potential conflicts of interest. Scientific Advisory Boards: Transdermal Cap Inc., Cleveland, OH; BeWell Global Inc., Wan Chai, Hong Kong; Hologenix Inc., Santa Monica, CA; LumiThera Inc., Poulsbo, WA; Vielight, Toronto, Canada; Bright Photomedicine, São Paulo, Brazil; Quantum Dynamics LLC, Cambridge, MA; Global Photon Inc., Bee Cave, TX; Medical Coherence, Boston, MA; NeuroThera, Newark, DE; JOOVV Inc., Minneapolis-St. Paul, MN; AIRx Medical, Pleasanton, CA; FIR Industries Inc., Ramsey, NJ; UVLRx Therapeutics, Oldsmar, FL; Ultralux UV Inc., Lansing MI; Illumiheal & Petthera, Shoreline, WA; MB Lasertherapy, Houston, TX; ARRC LED, San Clemente, CA; Varuna Biomedical Corp. Incline Village, NV; Niraxx Light Therapeutics Inc., Boston, MA; Consulting; Lexington Int, Boca Raton, FL; USHIO Corp, Japan; Merck KGaA, Darmstadt, Germany; Philips Electronics Nederland B.V., Eindhoven, Netherlands; Johnson & Johnson Inc., Philadelphia, PA; Sanofi-Aventis Deutschland GmbH, Frankfurt am Main, Germany. Stockholdings: Global Photon Inc., Bee Cave, TX; Mitonix, Newark, DE.

The remaining authors declare that the research was conducted in the absence of any commercial or financial relationships that could be construed as a potential conflict of interest.

## Abbreviations

A3BS: alternative 3′ back-splicing
A5BS: alternative 5′ back-splicing
AGO: argonaute
ASCC: anal squamous cell carcinoma
BART: Bam HI-A region rightward transcript
circBART: EBV-derived circRNA
circE7: HPV-E7 derived circRNA
circRNAs: covalent closed circular RNAs or circular RNAs
CMV: cytomegalovirus
EBER: EBV-encoded RNA
EBV: Epstein Barr virus
EMT: epithelial-mesenchymal transition
HBV: hepatitis B virus
HCC: hepatocellular carcinoma
HDAg: hepatitis delta antigen
HDV: hepatitis D virus
HHV: human herpesvirus
HPV: human papillomavirus
HSV: herpes simplex virus
IAV: influenza A virus
IRES: internal ribosome entry site
KSHV: Kaposi’s sarcoma-associated herpesvirus
lncRNA: long noncoding RNA
MBL: mannose-binding lectin
NGS: next-generation sequencing
NPC: nasopharyngeal carcinoma
PAN: polyadenylated nuclear RNA
PEL: primary effusion lymphoma
RNA-seq: RNA-sequencing
rRNA: ribosomal ribonucleic acid
SA409: splice acceptor 409
SD226: splicing between splice donor 226
snRNA: small nuclear RNA
snRNPs: small nuclear ribonucleoproteins
TGEV: transmissible gastroenteritis virus
tRNA: transfer ribonucleic acid
VcircRNAs: virus-encoded circRNAs
VZV: varicella-zoster virus.
